# Integrated approaches to COVID-19 emergency response in fragile, conflict-affected and vulnerable settings: a public health policy brief

**DOI:** 10.1057/s41271-022-00383-5

**Published:** 2022-12-23

**Authors:** Olushayo Oluseun Olu, Joy Luba Lomole Waya, Sandra Bankss, Sylvester Maleghemi, Argata Guracha Guyo

**Affiliations:** World Health Organization COVID-19 preparedness and response team, Juba, Republic of South Sudan

**Keywords:** Sustainable policies, COVID-19 response, Fragile conflict-affected and vulnerable settings, Sub-Saharan Africa

## Abstract

In the absence of fully effective measures to prevent and treat COVID-19, the limited access to and hesitancy about vaccines, the prolongation of the on-going pandemic is likely. This underscores the need to continue to respond and maintain preparedness, preferably using a more sustainable approach. A sustainable management is particularly important in fragile, conflict-affected and vulnerable countries of sub-Saharan Africa given several peculiar challenges. This *Viewpoint* proposes policy options to guide transitioning from current COVID-19 emergency response interventions to longer-term and more sustainable responses in such settings. In the long term, a shift in policy from a vertical to a more effective approach should integrate response coordination, surveillance, case management, risk communication and operational support, among other elements, for better results. We call on public health policymakers, partners and donors to support full implementation of these policy options in a holistic manner to encompass all emerging public health threats.

## Key messages

- Emergence of new strains of the virus that caused COVID-19, the transmission dynamics of the disease and limited access to and hesitancy about vaccines create need for a sustainable long-term approach—especially in fragile, conflict-affected countries of sub-Saharan Africa.
- A shift in policy from a vertical emergency response to a more effective long-term approach should integrate response coordination, surveillance, case management, risk communication and operational support.
- South Sudan implemented such changes from early 2021, and the experience offers lessons for other countries with similar challenges.

## Introduction

The Corona Virus Disease 2019 (COVID-19) pandemic in sub-Saharan Africa defied all modelling projections of a massive and widespread epidemic on the continent [1]. Contrary to predictions, recent epidemiological and modelling data indicate lower COVID-19 death rates in sub-Saharan Africa than in the rest of the world [2]. Although recent epidemiological data point to a declining trend in the disease incidence in most parts of the world [3], this does not forestall possibility for another surge in cases [4]. In the absence of fully effective means to prevent and treat the disease and limited access to and hesitancy about COVID-19 vaccines on the continent, the long propagation of the current pandemic is likely [5]. This situation underscores the need to respond to outbreaks and maintain preparedness, albeit with a more integrated and sustainable approach. Initial stages of the pandemic justified the use of special measures such as establishment of vertical platforms for coordination of the response, surveillance, case management, risk communication and procurement of essential medicines, medical supplies and equipment to address an emerging crisis. But continuing this way is not sustainable due to the dearth of resources such as human resources and predictable funding in most sub-Saharan African countries. The developing countries of sub-Saharan Africa thus need different approaches to control the COVID-19 pandemic and other emerging and equally important public health threats [6].

## Need for a longer-term, integrative approach to COVID-19 response in fragile, conflict-affected and vulnerable settings

The World Health Organization (WHO) defines fragile, conflict-affected and vulnerable (FCV) countries as those experiencing situations of crises due to protracted emergencies, disruption of governance structures and/or armed conflict [7]. While the WHO nomenclature is slightly different from that of the World Bank Group which uses “violence” instead of “vulnerable”, the WHO classification of FCV countries largely depends on the World Bank Group list [8]. This list currently includes 37 countries out of which 18 are in sub-Saharan Africa.^1^ In the context of this article, the term “fragile” refers to countries who are in unstable situations while “vulnerable” refers to countries who are at risk of multiple public health emergencies and both terms are viewed as distinct.

FCV countries of sub-Saharan Africa face peculiar challenges, such as pervasively weak health system, vulnerability to multiple public health emergencies, increased demand for healthcare services in the face of disrupted or weak routine healthcare delivery systems and inadequate health funding among others. The COVID-19 pandemic has further aggravated these challenges.

First, the COVID-19 pandemic overwhelmed already compromised health systems in these countries, rapidly depleting critical human, financial and material resources and adversely disrupting the delivery of essential health services. In South Sudan, a special survey estimated health workforce density (doctors, nurses and midwives) at 0.76 per 1000 population [unpublished data from the South Sudan Services Availability and Readiness Survey, 2018]—far below the Sustainable Development Goals (SDG) threshold of 4.45 per 1000 population for making progress towards Universal Health Coverage [9]. Within the countries of the WHO African Region, mainly in Sub-Saharan Africa, health workforce density is as low as 1.55 per 1000 population, with only four countries in the region (Mauritius, Seychelles, South Africa and Namibia) above the 4.45 threshold. FCV countries, such as Chad and Central African Republic, are among those with the lowest levels, fewer than 0.5 health workers per 1000 population [10]. For 2020, South Sudan’s Humanitarian Response Plan estimated additional funding needed for COVID-19 at USD 150 million, or 8% of the original amount of $1.9 billion allocated for the overall humanitarian response for the year [11]. For the same year, the South Sudan Health Sector Strategic Plan listed the amount needed for the entire health sector routine programming at $457 million—largely designated for development interventions in the health care sector [unpublished data from the South Sudan Health Sector Strategic Plan 2017–2022]. Based on the 2020 Humanitarian Response Plan, the additional funds needed for COVID-19 response in 2020 alone, was 33% of the entire sector budget for the year.

Second, these countries continue to experience multiple and recurrent emergencies arising from conflicts, natural disasters such as droughts, floods and disease outbreaks, including of wild polio virus (and its vaccine derived variant), yellow fever, cholera, measles, meningitis, among others, due to prevailing poor living conditions and weak health systems. Third, the social, economic and cultural impacts of the pandemic have been particularly severe in these countries, including aggravation of poverty through disruption of livelihoods and of societal structures. Fourth, the current approach to COVID-19 epidemic response, which relies on vertical systems and structures is resource intensive and not sustainable.

We agree with Ali et al. on the need for longer-term, sustainable, cost-effective and integrated responses to the outbreaks in FCV settings [12]. The aims of such an approach are three: to enhance global health security, ensure health system recovery and strengthen health system resilience [13, 14]. We propose policy options to guide transition from the initial emergency public health responses to the COVID-19 pandemic to humanitarian and development programming. We present a case study of South Sudan where health system challenges are enormous, and the complexity of its humanitarian crisis is among the most challenging in sub-Saharan Africa. We next summarize policy options and principles to guide implementation of more sustainable approaches. Then we discuss implications of the changes and summarize early lessons from implementation in South Sudan to inform the transition from emergency (vertical) COVID-19 responses to longer-term approaches integrating COVID -19 into routine care and structures in similar settings.

## South Sudan as a case study: shifting from vertical to an integrated COVID-19 response

South Sudan, the world's youngest nation, grapples continuously with armed conflicts, natural disasters and chronic underdevelopment. Together these have decimated the country's health system, leaving inadequate availability of good quality, sustainable and affordable health services. The country reported its first case of COVID-19 on 5 April 2020 [15], and by 26 October 2022, it had reported 17,780 confirmed cases and 138 deaths [16]. As elsewhere in sub-Saharan Africa, the overall reported number of cases and deaths amounted to far fewer than anticipated. Limited surveillance and laboratory capacity could have contributed to the low counts.

At the onset of the outbreak in April 2020, the country, with the support of its partners such as United Nations agencies, national and international non-governmental organizations and the academia, established a vertical outbreak response system. It included a COVID-19 national incident management system and national taskforce, and a COVID-19 surveillance system with alerts, rapid response, case investigation, contact tracing, mortality surveillance and Points of Entry (PoE) components. The Ministry of Health rapidly adapted existing capacity for laboratory diagnosis of Ebola and other viral diseases for COVID-19 diagnosis while establishing case management centres in all the ten States of South Sudan. South Sudan also established structures for COVID-19 risk communication and community engagement and produced communication materials such as posters, billboards, television and radio messages among others. It also procured COVID-19 equipment, medicines and supplies through a dedicated global COVID-19 operational support and logistic platform. And, in the latter part of the response, the country introduced a COVID-19 vaccination strategy of campaigns.

These interventions proved effective, but not sustainable. Special measures to address the emergence of COVID-19 stressed South Sudan’s pervasively weak health system as it struggled simultaneously to deal with other equally important needs, including outbreaks of cholera, measles, meningitis, yellow fever, among others, as well as perennial flooding. Establishing vertical incident management systems for each public health event proved impracticable and not cost-effective given the unpredictability of longer-term funding and dearth of human resources. Domestic resources remained grossly inadequate for meeting these needs. Thus, in the last quarter of 2020, the WHO COVID-19 response team worked with stakeholders in the country to develop the options to improve policies that we present below.

## Policy options and key messages for integrating COVID-19 response into FCV countries’ health and humanitarian systems

Our objective in presenting these options is to assist FCV countries to integrate on-going vertical COVID-19 response interventions into existing humanitarian response or health sector-wide development coordination systems’ plans and programming. Table 1 presents in detail the policy shifts we propose. There, and below, we refer to “pillars” that signify the internationally proposed components of the COVID-19 response system [17].

**Table 1:** Specific policy options for transitioning on-going COVID-19 response to a longer-term and integrated approach in FCV countries

COVID-19 response pillar	On-going vertical emergency response activities	Proposed long-term and integrated response activities
Coordination	Multisectoral National Task Forces (NTF), usually under the Office of the President or Prime Minister Public Health Emergency Operations Centres (PHEOC) provide the structure and framework for COVID-19 response operations In-house coordination mechanisms among international organizations (for instance in South Sudan, a COVID-19 leadership group was created as a sub-group of the Humanitarian Country Team (HCT). This group had an operational component)	Ministries of Health should: Strengthen coordination platforms such as the Health Sector Working Group (HSWG), the overarching health coordination platform to use for oversight and leadership of current and future outbreak response activities Co-opt representatives from relevant sectors into the HSWG to ensure a whole-of-government approach to health and emergencies, with a clear accountability framework for all relevant stakeholders Align and, where feasible integrate the COVID-19 National Response Plan elements into existing strategies and plans for the various response pillars Humanitarian and development partners should: Retract COVID-19 specific international organization coordination fora into previous regular humanitarian fora such as the HCT and Inter-Cluster Coordination Group Integrate coordination of the COVID-19 response into existing Humanitarian Cluster mechanisms such as the Health Cluster, other relevant Humanitarians Clusters and the Inter-Cluster Coordination mechanism (WASH, Education, Logistic Clusters among others) and the Emergency Preparedness and Response forum and use existing thematic Technical Working Groups of the HSWG and Humanitarian Clusters to address specific activities Continue partner support to the PHEOC and COVID-19 coordination response mechanism while building local capacity, scaling down and transitioning to minimum capacities, and advocating for increased and sustained government funding for operations of the PHEOCs
Surveillance	Presence of various COVID-19 surveillance teams for alerts, rapid response, case investigation, contact tracing and national mortality surveillance There is a COVID-19 national database which is housed in the PHEOC	Ministries of health should: Adapt and retract COVID-19 surveillance components into the existing national surveillance system, such as the IDSR and EWARN Maintain and expand existing Rapid Response Teams (RRTs) structure to continue investigating COVID-19 clusters and other public health alerts and build capacity for all-hazard intervention Strengthen health facility level, sentinel site and community-based surveillance for all public health threats including COVID-19 Use existing data management and reporting resources at all levels including the District Health Information System Build capacity for digital data collection and reporting using existing digital health data collection tools such as Open Data Kits (ODK) and strengthen data analysis capacity at the national and subnational levels to enable evidence-based decision making as the pandemic and other public health events evolve
Points of Entry (PoE)	COVID-19 specific syndromic surveillance at the PoE at the air and land borders through monitoring the exposure, travel history and body temperature of passengers	Ministries of health should: Implement the PoE component of the NAPHS, by establishing functional Port Health Units (national and sub-national) at major airports and land borders to integrate IPC measures, epidemiologic surveillance, vaccination and other relevant health services as per the International Health Regulations Identify priority locations to establish Port Health Units, develop the necessary Port Health infrastructure, build capacity of health staff and provide tools and equipment
Laboratory diagnosis and management	A decentralized COVID-19 testing system largely supported by private and public laboratories using PCR and GeneXpert testing strategies	Ministries of health should implement the laboratory component of the NAPHS by: Strengthening in-country laboratory network to enable testing of all major notifiable or reportable diseases including COVID-19 at the national and subnational levels Build capacity of national and sub-national laboratory technical human resources to test for emerging and re-emerging priority diseases to fast track and augment surveillance and response efforts as required Develop and implement a long-term National Laboratory Testing Strategy (or Guideline) Establish an efficient in-country laboratory logistics network Define and utilize lab-quality checks for consistency and quality of results
Case management and continuity of essential health services	Home-based isolation and management of uncomplicated COVID-19 cases Facility-based management of severe COVID-19 in specifically designated facilities Monitoring of utilization of essential services through reviewing of tracer indicators such as outpatient department utilization, Pentavalent3 and Measles immunization coverage, antenatal 1 and 4 coverage, and skilled birth attendance trends from the second edition of the District Health Information System (DHIS2) for national and sub-national levels	Ministries of health should: Integrate COVID-19 into care at facility levels. COVID-19 triage, case management and IPC in health facilities to be delivered as an integrated package as part of on-going essential health services with both static and mobile humanitarian services delivery mechanisms Provide all health care workers with capacity and tools to conduct and maintain triage, implement facility-based surveillance and manage cases of COVID-19 like illness and other infectious diseases Convert the established COVID-19 isolation facilities or wards into permanent isolation units for isolation of other infectious diseases and designate isolation units in other secondary and tertiary health care facilities Integrate COVID-19 and other infectious diseases triage and case management into routine operations of health facilities Continue monitoring the utilization of essential services at national and sub-national levels through monthly review of trends of tracer indicators Mount appropriate response upon detection of disruption of utilization of essential health services; address causes identified for low service utilization
Infection prevention and control (IPC)	Implementation of vertical COVID-19 specific IPC activities in line with the COVID-19 Standard Operating Procedures and technical guidelines	Ministries of health should: Establish a comprehensive and integrated IPC approach at health care facilities for COVID-19 and other infectious and epidemic prone diseases, integrated into the overall essential health services delivery structures by leveraging support from implementing partners operating the health facilities. This approach includes: Establishment of triage units in health facilities Training of health workers on IPC Regularly providing sufficient quantities of IPC related supplies (including personal protective equipment for health workers) and water, sanitation and hygiene (WASH) supplies Conduct regular IPC/WASH service assessments and address identified gaps Establish National, State and County level IPC committee structures to support implementation, supervision, monitoring and evaluation of IPC activities Develop and implement comprehensive national IPC strategies
Risk Communication and Community Engagement (RCCE)	Vertical COVID-19 RCCE structure	Ministries of health should: Retract COVID-19 specific RCCE activities and integrate them into existing, routine technical working groups such as the Behaviour Change Communication of the HSWG Integrate COVID-19 key messaging into: National health promotion strategies and messaging Routine health education and promotion activities and information, education and communication material development Routine public health promotion activities and key messaging conducted by Community Health Workers and existing partner community mobilisers networks Integrate COVID-19 into risk communication and community engagement training packages Establish a monitoring mechanism to track and address all rumours associated with infectious disease outbreaks and notifiable/reportable diseases
Operation support and logistics	Specific system for COVID-19 operation, logistics and supply chain management system (mainly by international organizations) was established	Humanitarian partners should support Ministries of health to: Integrate the COVID-19 logistics and supply chain management into the National supply chain management system for essential medicines and medical supplies Procure COVID-19 supplies through the Emergency Core Pipeline System through the Health Cluster Strengthen the capacity of logistics personnel at national and sub-national levels through training and provision of relevant tools on stock management, inventory and dispatch Develop and disseminate Standard Operating Procedures for inventory and stock management for the Ministry of Health Establish emergency preparedness stock and simulate emergency preparedness planning for potential emergencies Continue to provide operations and logistics support through partners and the Logistics cluster, cognizant of the country context regarding access and security
Vaccine management	Vertical COVID-19 vaccination system	Ministries of health through their national immunization programmes should: Integrate COVID-19 vaccination into the routine immunization system Strengthen the routine immunization systems to include COVID-19 vaccination requirements (cold chain equipment, data reporting tools, communication, social mobilization etc.) Integrate COVID-19 into mass vaccination campaigns where feasible

In summary, we recommend that countries:

- Move the coordination pillar of the response into the existing national coordination system, such as the health sector working group, the health cluster and inter-cluster coordination mechanisms.
- Integrate the surveillance pillar of the COVID-19 response into existing, routine national public health surveillance systems such as the Integrated Disease Surveillance and Response (IDSR) and the Early Warning Alert and Response Network (EWARN). The IDSR is the WHO recommended routine system for collection and management of disease data at the country level; the EWARN is specifically used in humanitarian settings such as South Sudan.
- Expand the current PoE pillar (comprising  the COVID-19 surveillance at the land and air borders) to incorporate all aspects of the more robust port health services and integrate them into the National Action Plans for Health Security (NAPHS). The laboratory component of the pandemic requires a longer-term approach as part of a broader national laboratory strategic plan aimed at addressing the laboratory diagnosis of multiple pathogens.
- Deliver the case triage, isolation, management, Infection Prevention and Control (IPC), and continuity of essential services pillars as an integrated package of on-going essential health services. That is, incorporate these pillars into existing service delivery components of the health sector such as the static health facilities and mobile humanitarian health services delivery mechanisms.
- Integrate the Risk Communication and Community Engagement (RCCE) pillar into the routine national health education and promotion interventions and communication materials.
- Combine the operational support and logistics system with the national supply chain management system and the emergency core pipeline system which is managed by the health cluster.
- Integrate the vaccine management pillar into the routine immunization system.

We also propose a few principles to guide implementation of the policy shifts:

- Maintain government leadership through their ministries of health and continuing collaboration with relevant partners. Political commitment at the highest level of government is central to a smooth transition—particularly for effective coordination among stakeholders, to ensure buy-in, accountability and harmonization of the transition process with national health priorities and interventions.
- Integrate all pillars of the COVID-19 national response plans into existing health sector plans to ensure comprehensive implementation and facilitate progress towards Universal Health Coverage. (These include the NAPHS, national health system recovery and stabilization plan, the humanitarian response plan and the health sector strategic plan.)
- Establish a horizontal approach to emergency response for all emerging public health threats to prevent stretching the already limited resources.
- Build health system resilience and global health security as fundamental elements for effective emergency preparedness and response. Emergency response should provide opportunities to strengthen the health system using existing funding, such as funds for the COVID-19 response, to fill critical gaps in the health system. This means adopting a health system strengthening approach during all phases of the COVID-19 response.
- Emphasize continuity of all essential health services, those often disrupted during pandemics.
- Maintain basic preparedness capacity to respond to resurgence in COVID-19 cases by maintaning minimum capacities for each of the pillars of the COVID-19 response within the broader framework of the NAPHS.

## Implications of shifting policies and implementing the changes

Countries should anticipate and plan responses to actions, and to reactions, both negative and positive that such policy shifts may attract. Stakeholders often resist policy changes due to personal or organizational interests, competition for scarce resources or unwillingness to change traditional methods of operation. The resistance may deepen if communication of a new policy to the stakeholders is inadequate. Poor coordination may lead to duplication and gaps in emergency response efforts. However, integration of COVID-19 emergency response efforts with routine service delivery could improve cost-effectiveness, harmonization and alignment of humanitarian response interventions with national health priorities and strengthen health system resilience.

We recommend a proactive and systematic approach to address these challenges, emphasizing

- Strong advocacy and communication with all stakeholders to ensure common understanding and buy-in to the policy shifts.
- Wide dissemination of documents explaining policy shifts to the relevant target audiences at national and sub-national levels.
- Development of frameworks that will be necessary to effectively plan, supervise, monitor and evaluate the policy shifts process. This will require well-defined baselines and targets.

Furthermore, countries should watch for lessons from implementation of the policy shifts, document them systematically and use the observations as evidence to improve the overall process.

## Lessons learned from early phase implementation in South Sudan and recommendations

South Sudan commenced pilot testing of these policy shifts in the first quarter of 2021. Lessons we report here for use in other FCV settings had emerged as of October 2022. We observed and recorded varied levels of success. We saw good progress in surveillance, case management and continuity of essential health services, IPC and vaccine management response efforts. We observed little progress, however, in coordination, risk communication and community engagement and in operation support and logistics. Anecdotal evidence indicated progress for those pillars with strong routine systems. For instance, South Sudan built on its fairly robust routine surveillance system (the IDSR and EWARN) to transition the surveillance component of the COVID-19 response. Similarly, the IPC pillar benefitted from recent development and on-going implementation of a national IPC strategic plan and large-scale training of healthcare workers on IPC. The little progress in coordination may be attributed to the weak capacity of the health sector working group, the main focal point for coordination for all health activities in the country. Activities in the operations support and logistic pillar improved little, likely due to the weak national supply chain management system.

The vaccination systems, structures and human resources used for the pandemic relied heavily on the existing elements of routine immunization, including the cold chain equipment and vaccinators. However, delivery of COVID-19 vaccination as campaigns added stress to a weak system. It was the same health workers who provide routine immunization services who added COVID-19 vaccination campaigns to their responsibilities. We recommend addressing this challenge by using COVID-19 vaccination funds to strengthen routine immunization structures including cold chain equipment, supplies and staffing, and by revising the national routine immunization policy, strategy, schedule and mass campaigns to include COVID-19.

These lessons reinforce the importance of using emergency resources to strengthen routine health system elements where feasible and vice versa.

## Conclusion

Although the COVID-19 pandemic trend has been declining recently in many parts of the world, emergence of new variants may result in resurgence if countries do not sustain effective preparedness and response capacities. The resource-intensive nature of responding to COVID-19 outbreaks and health system challenges in FCV settings both reinforce the importance of pursuing integrated and cost-effective COVID-19 outbreak responses. Early adoption and implementation of the approaches we recommend have several advantages: opportunities to strengthen the health system and global health security capacity to quell resurgence of the COVID-19 pandemic and other emerging public health threats, now including Monkeypox which has recently been renamed as mpox by WHO. We call on all public health policy makers, development and humanitarian partners, along with the donor community, to support full implementation of the proposed policy shifts in all FCV settings and to do so holistically, to encompass all emerging public health threats. For South Sudan, where implementation is underway, we recommend on-going supervision, monitoring, evaluation and systematic documentation of lessons from the process––to benefit South Sudan and all who can learn from their experience.

## Data Availability

Data sharing is not applicable to this article as no datasets were generated or analysed during the current study.

## Glossary

**Table:**

Development partners	These are partners who specifically focus on supporting national authorities to implement longer-term development interventions at the country level. They include donors, UN agencies, international and national non-governmental agencies
Development programming	Refers to the activities involved with the planning, implementation, supervision, monitoring and evaluation of longer-term development interventions at the country level
Early warning, alert and response network	This is a network for diseases surveillance and response in humanitarian settings where routine systems such as the IDSR are unavailable or underperforming. Its focus is on early detection of and rapid response to disease outbreaks or other public health emergencies in internally displaced persons/refugee camps and conflicted-affected zones
Emergency core pipeline	A system for forecasting, procurement, storage and distribution of emergency supplies such as emergency medical kits, food, non-food items etc. in humanitarian/FCV settings. The health component is managed by the health cluster
Health cluster	The humanitarian cluster designated to coordinate public health interventions during humanitarian crises. It is led by WHO globally and at the country level
Pooled funds	This is a mechanism used by donors to pool their financial contributions into a single fund to support humanitarian or development activities at the country level. Such funds are usually managed by a single administrative agent to ensure efficiency. Country-based pooled funds support humanitarian interventions at the country level and is managed by the United Nations Office for Coordination of Humanitarian Affairs. A few FCV countries have other pooled funds mechanisms such as the Health Pooled Funds that support routine health services delivery in South Sudan
Health sector working group	This is a government-led coordination body that is responsible for the overall coordination of health activities at the country level. It is usually chaired by the Ministry of Health and comprise several thematic technical working groups depending on the country context. In general, health sector working groups are longer-term coordination mechanisms which focus on development work while the health cluster is short-term and focuses on emergencies
Humanitarian clusters	This is an emergency coordination system that is activated by the inter-agency standing committee during humanitarian crises when existing national coordination mechanisms are overwhelmed and unable to respond to the humanitarian needs. They are groups of organizations who support planning, implementation, supervision, monitoring and evaluation of humanitarian interventions in specific areas of work. Globally, there are 11 designated humanitarian clusters including the health cluster
Humanitarian health services	These are health services that are primarily focused on crises affected populations such as displaced populations, vulnerable groups such as women, children, the disabled, elderly persons, migrants etc
Humanitarian partners	These are partners who primarily support national authorities to deliver humanitarian assistance at the country level. They include donors, UN agencies, international and national non-governmental agencies
Humanitarian programming	Refers to the activities involved with the planning, implementation, supervision, monitoring and evaluation of humanitarian assistance at the country level
Integrated disease surveillance and response system	This is a strategy for strengthening routine public health surveillance and response at the national level which was adopted by all Member States of the WHO African Region in 1998. It is a vehicle for achieving the core capacities of the 2005 International Health Regulations in the African Region. It complements the Early Warning, Alert and Response Network which is used in humanitarian settings
Inter cluster coordination mechanism	This a platform under which all humanitarian clusters work together at the country level in a well-coordinated, efficient and effective manner. It comprises the Leads of all humanitarian clusters in a country (including the health cluster) and is coordinated by the United Nations Office for the Coordination of Humanitarian Affairs
National action plan for health security	This is a multi-year national plan (usually for five years) which is aimed at strengthening national health security through accelerating the implementation of the 2005 International Health Regulation core capacities at the country level. It includes priority actions for ensuring preparedness for, response to and recovery from public health emergencies within the framework of the 2005 International Health Regulations
Points of entry	These are the land, air and sea borders of a country where designated health screening activities takes place as part of the 2005 International Health Regulations core capacities strengthening
Public health emergency operations centre	This is a physical or virtual space where all designated emergency functions are performed during a public health emergency. It aims to effectively coordinate emergency information, resources and operations
Static health care facilities	These are fixed health care delivery units as opposed to mobile health care delivery units
Tracer indicators	These are health indicators such as immunization coverage, antenatal and skilled birth attendance, outpatient services utilization etc. which are used as proxies to monitor the continuity of essential health services during a public health emergency. These indicators are being widely used during the COVID-19 pandemic to monitor health services

## Abbreviations

COVID-19: Coronavirus Disease 2019
EWARN: Early Warning Alert and Response Network
FCV: Fragile, Conflict-affected and Vulnerable
IDSR: Integrated Diseases Surveillance and Response
IPC: Infection Preventions and Control
NAPHS: National Action Plan for Health Security
PHEOC: Public Health Emergency Operations Centre
PoE: Points of Entry
RCCE: Risk Communication and Community Engagement
WHO: World Health Organization

## Footnotes

1 Sub-Saharan African countries listed as fragile, conflicted-affected and vulnerable by the World Bank: Burundi, Cameroon, Central Africa Republic, Chad, Comoros, Democratic Republic of Congo, Eritrea, Ethiopia, Guinea Bissau, Mali, Mozambique, Niger, Nigeria, Somalia, South Sudan, Sudan, Republic of Congo and Zimbabwe.
